# Diurnal Variation of Urinary Fabry Disease Biomarkers during Enzyme Replacement Therapy Cycles

**DOI:** 10.3390/ijms21176114

**Published:** 2020-08-25

**Authors:** Michel Boutin, Pamela Lavoie, Iskren Menkovic, Amanda Toupin, Mona Abaoui, Maha Elidrissi-Elawad, Marie-Françoise Arthus, Carole Fortier, Claudia Ménard, Bruno Maranda, Daniel G. Bichet, Christiane Auray-Blais

**Affiliations:** 1 CIUSSS de l’Estrie-CHUS, Université de Sherbrooke, Centre de Recherche-CHUS, 3001, 12th Avenue North, Sherbrooke, QC J1H 5N4, Canada; Michel.Boutin2@USherbrooke.ca (M.B.); pamela.lavoie@usherbrooke.ca (P.L.); Iskren.Menkovic@USherbrooke.ca (I.M.); amanda.toupin@usherbrooke.ca (A.T.); mona.abaoui@usherbrooke.ca (M.A.); Maha.Elidrissi.Elawad@USherbrooke.ca (M.E.-E.); bruno.maranda@usherbrooke.ca (B.M.); 2 CIUSSS du Nord-de-l’Île-de-Montréal, Hôpital du Sacré-Coeur de Montréal, Clinical Research Unit, 5400 Gouin Blvd West, Montreal, QC H4J 1C5, Canada; marie-francoise.arthus@umontreal.ca (M.-F.A.); C-Fortier@crhsc.rtss.qc.ca (C.F.); claudia.menard@crhsc.rtss.qc.ca (C.M.); daniel.bichet@umontreal.ca (D.G.B.); 3 Department of Medicine Pharmacology and Physiology, Université de Montréal, 2900 Édouard-Montpetit Blvd, Montreal, QC H3T 1J4, Canada

**Keywords:** Fabry disease, diurnal variation, globotriaosylceramide, globotriaosylsphingosine, mass spectrometry, glycosphingolipids

## Abstract

Fabry disease is an X-linked lysosomal storage disorder caused by mutations in the *GLA* gene encoding the α-galactosidase A enzyme. This enzyme cleaves the last sugar unit of glycosphingolipids, including globotriaosylceramide (Gb_3_), globotriaosylsphingosine (lyso-Gb_3_), and galabiosylceramide (Ga_2_). Enzyme impairment leads to substrate accumulation in different organs, vascular endothelia, and biological fluids. Enzyme replacement therapy (ERT) is a commonly used treatment. Urinary analysis of Gb_3_ isoforms (different fatty acid moieties), as well as lyso-Gb_3_ and its analogues, is a reliable way to monitor treatment. These analogues correspond to lyso-Gb_3_ with chemical modifications on the sphingosine moiety (−C_2_H_4_, −C_2_H_4_+O, −H_2_, −H_2_+O, +O, +H_2_O_2_, and +H_2_O_3_). The effects of sample collection time on urinary biomarker levels between ERT cycles were not previously documented. The main objective of this project was to analyze the aforementioned biomarkers in urine samples from seven Fabry disease patients (three treated males, three treated females, and one ERT-naïve male) collected twice a day (morning and evening) for 42 days (three ERT cycles). Except for one participant, our results show that the biomarker levels were generally more elevated in the evening. However, there was less variability in samples collected in the morning. No cyclic variations in biomarker levels were observed between ERT infusions.

## 1. Introduction

Fabry disease (FD) (OMIM no. 301500) is an X-linked monogenic lysosomal storage disorder. While males are often more affected than females, some females can be as severely affected as males due to X-chromosome inactivation [1]. The main clinical features of FD are cardiac hypertrophy, progressive renal deficiency, and increased risk of ischemic strokes. Other signs and symptoms include acroparesthesia, hypohidrosis, gastrointestinal involvement, angiokeratomas, cornea verticillata [2], and vascular tortuosities of the upper eyelid [3]. FD is caused by mutations in the *GLA* gene encoding the α-galactosidase A enzyme (EC 3.2.1.22). The function of this glycoside hydrolase is to cleave the terminal α-galactosyl moieties from glycoproteins or glycolipids [1]. The deficiency of the enzyme leads to the accumulation of substrates such as globotriaosylsphingosine (lyso-Gb_3_), galabiosylceramide (Ga_2_), and globotriaosylceramide (Gb_3_) in cells [4], tissues [5], and biological fluids [6,7,8,9]. Different untargeted metabolomic studies performed in our laboratory with urine and plasma specimens from FD patients identified different analogues of lyso-Gb_3_ [10,11], as well as analogues/isoforms of Ga_2_ [12] and Gb_3_ [13], as FD biomarkers. The analogues correspond to molecules with modified sphingosine moieties, whereas the isoforms correspond to molecules with different fatty acid chains. As for lyso-Gb_3_ in urine, the following modifications on the sphingosine moiety were detected: −C_2_H_4_ (−28 Da), −C_2_H_4_+O (−12 Da), −H_2_ (−2 Da), −H_2_+O (+14 Da), +O (+16 Da), +H_2_O_2_ (+34 Da), and +H_2_O_3_ (+50 Da) [10]. In fact, most of the lyso-Gb_3_ analogues in urine are more abundant than lyso-Gb_3_ itself. Previous results showed that FD children with the late-onset cardiac variant *p*.N215S mutation presented normal urinary levels of lyso-Gb_3_, but abnormal levels of some lyso-Gb_3_ analogues [14]. Similarly, five patients with the *p*.N215S mutation presented abnormal levels of methylated Gb_3_ isoforms, but normal levels of non-methylated Gb_3_ isoforms [7]. Moreover, the analysis of urine samples from FD patients with the late-onset IVS4+919G > A cardiac variant mutation prevalent in Taiwan revealed that some lyso-Gb_3_ analogue levels had a positive association with the left-ventricular mass index and/or the Mainz Severity Score Index [15]. Chaperone therapy with migalastat (Galafold, Amicus Therapeutics) is now available for patients with amenable mutations [16]. However, the most common treatment for FD remains ERT [17], which consists of bi-weekly intravenous infusions of recombinant α-galactosidase A such as agalsidase-alpha at 0.2 mg/kg (Replagal, Shire/Takeda Pharmaceutical) or agalsidase-beta at either 1.0 or 0.3 mg/kg (Fabrazyme, Sanofi Genzyme). A gene therapy clinical trial for FD is also ongoing [18]. The analysis of biomarkers in urine is a method of choice for monitoring the response of FD patients to ERT since sample collection is non-invasive. However, to the best of our knowledge, there are no published studies investigating the diurnal variation of urinary biomarkers and their fluctuation between ERT infusions. The main objectives of this research project were, thus, to evaluate if the urinary FD biomarker concentrations show statistically significant differences when sampled in the morning compared to the evening, and if the levels fluctuate periodically between ERT infusions using bi-daily monitoring over three ERT cycles (42 days).

## 2. Results

### 2.1. Biomarker Measures according to the Collection Time

#### 2.1.1. Mean Measured Biomarker Levels

**Figure 1:**
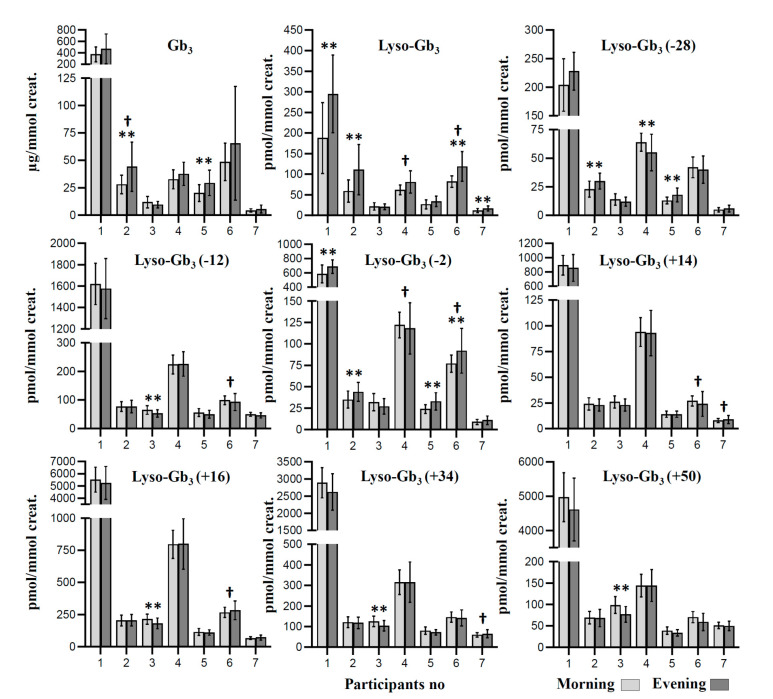
Mean concentration of Fabry biomarkers for three enzyme replacement therapy (ERT) cycles (*n* = 42 days) at two different collection times in seven study participants. All participants were under ERT, except participant no. 1 who never received treatment. Patients 1–4 were males, whereas patients 5–7 were females. For lyso-Gb_3_ analogues, the mass difference in Da compared to lyso-Gb_3_ is indicated in brackets. ** corrected *p*-value ≤ 0.001 for the paired-sample *t*-test; † corrected *p*-value ≤ 0.001 for Levene’s test for equality of variances.

Raw data are available in Supplemental Table S1. FD biomarkers are expressed as mean measured levels for each collection time point (Figure 1). Paired-sample *t*-tests were used to determine whether there was a statistically significant mean difference between the biomarker concentrations measured in specimens collected in the morning compared to specimens collected in the evening. Results are presented in Table 1 for each participant, along with a mean ± one standard deviation for each collection time. Results show that the measured concentrations might be influenced by the collection time for some biomarkers. Our results show a statistically significant mean difference for lyso-Gb_3_ levels in four participants (1, 2, 6, and 7), where concentrations were higher in urine specimens collected in the evening compared to those collected in the morning. Similar results were obtained for lyso-Gb_3_ analogue (−2 Da) in participants 1, 2, 5, and 6, and for Gb_3_ in participants 2 and 5. Regarding lyso-Gb_3_ analogues (−12 Da), (+14 Da), (+16 Da), (+34 Da), and (+50 Da), there were no mean differences in the measured levels based on the collection time, except for patient 3, where the measured values had a tendency to be higher in morning specimens. Finally, there were mixed results for the lyso-Gb_3_ analogue (−28 Da), where the measured values were higher in the evening for participants 2 and 5, but higher in the morning for participant 4.

#### 2.1.2. Variance of Biomarker Measures

Levene’s test for equality of variances was used to verify the homogeneous variance of biomarker levels when urine specimens were collected either in the morning or in the evening. Results are shown in Table 2 for each collection time, along with relative standard deviations (RSDs) (*n* = 42 days). In general, RSDs were higher for specimens collected in the evening. The variance was equivalent for the morning and evening specimens for each biomarker according to Levene’s test for participants 1, 3, and 5. However, Gb_3_ levels had more variance in evening specimens for participant 2, while lyso-Gb_3_ and the analogue (−2 Da) levels had more variance in evening specimens for participant 4. For participant 6, our results show that five biomarkers (lyso-Gb_3_, and analogues (−12 Da), (−2 Da), (+14 Da), and (+16 Da)) had a higher variance for specimens collected in the evening. The same tendency was observed for lyso-Gb_3_ analogues (+14 Da) and (+34 Da) for participant 7.

### 2.2. Longitudinal Follow-Up

#### 2.2.1. Untreated Fabry Disease Participant

**Figure 2:**
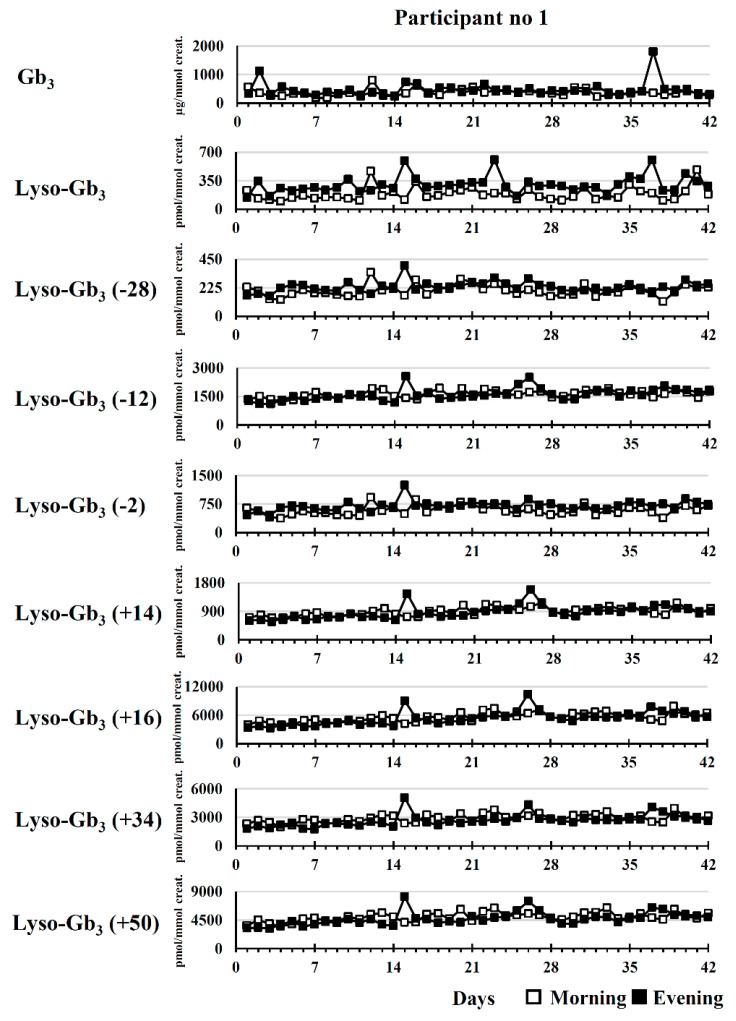
Longitudinal variation (*n* = 42) of Fabry biomarker levels in a 30-year-old untreated male (no ERT) (participant no. 1). For lyso-Gb_3_ analogues, the mass difference in Da compared to lyso-Gb_3_ is indicated in brackets.

The longitudinal biomarker follow-up of participant no. 1 was useful to evaluate the variability of the measured biomarker concentrations in urine specimens from an untreated Fabry patient. Longitudinal follow-ups are shown in Figure 2. According to RSDs, the dispersion of the measured concentrations (*n* = 42) was higher for lyso-Gb_3_ (morning RSD: 46%; evening RSD: 32%) and Gb_3_ (morning RSD: 34%; evening RSD: 56%) compared to lyso-Gb_3_ analogues with RSDs ≤ 23% in urine specimens collected in the morning, and RSDs ≤ 26% in urine specimens collected in the evening. 

#### 2.2.2. Participants Treated with ERT

Considering that biomarker levels had equivalent or lower variance in urine specimens collected in the morning compared to urine specimens collected in the evening, results of longitudinal biomarker follow-ups in treated males and females are shown in specimens collected in the morning only. 

##### Male Fabry Disease Participants

**Figure 3:**
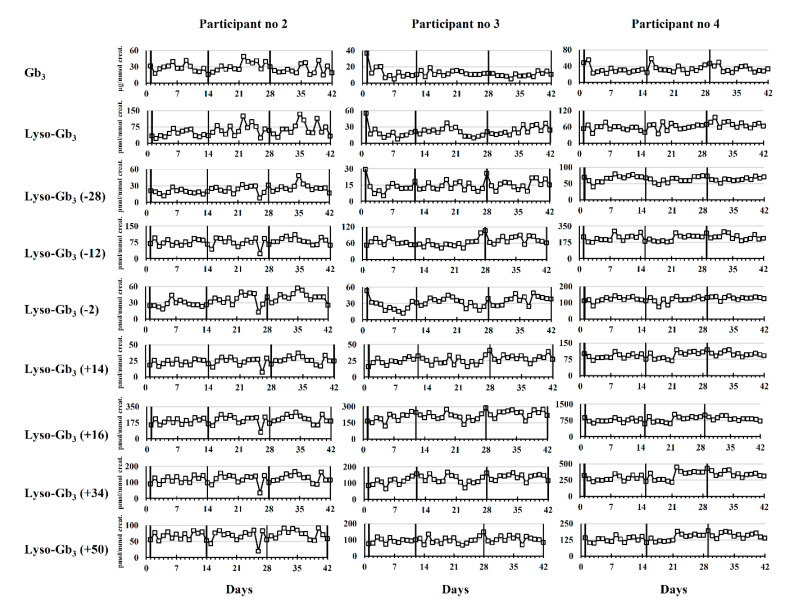
Longitudinal variation (*n* = 42) of Fabry disease biomarker levels during three ERT cycles in male patients. Results are shown for morning urine collection only. For lyso-Gb_3_ analogues, the mass difference in Da compared to lyso-Gb_3_ is indicated in brackets. Vertical lines indicate the time points where Fabry patients received their ERT infusions.

Longitudinal biomarker follow-ups are shown in Figure 3 for ERT-treated male participants 2, 3, and 4. 

Visual inspection of longitudinal follow-ups in Figure 3 suggests that, in general, the biomarker concentrations were stable in male patients during the study and oscillated around a mean biomarker value. In fact, no clear pattern of cyclic variation was observed in relation to ERT infusions. A visual examination of longitudinal follow-ups also showed that lyso-Gb_3_ analogues (−12 Da), (+14 Da), (+16 Da), (+34 Da), and (+50 Da) had similar profiles.

##### Female Fabry Disease Participants

**Figure 4:**
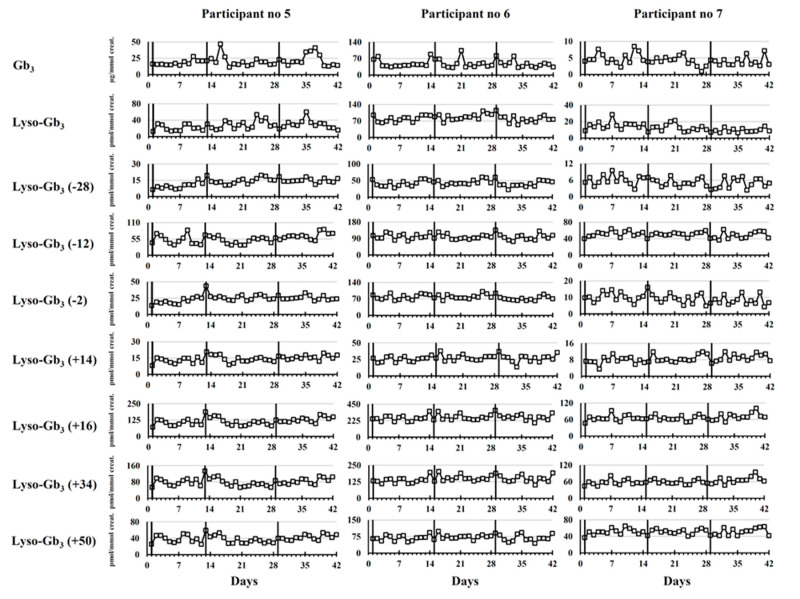
Longitudinal variation (*n* = 42) of Fabry disease biomarker levels during three ERT cycles in female patients. Results are shown for morning urine collection only. The vertical lines indicate the time points where Fabry patients received their ERT infusions. For lyso-Gb_3_ analogues, the mass difference in Da compared to lyso-Gb_3_ is indicated in brackets.

Longitudinal biomarker follow-ups are shown in Figure 4 for female participants 5, 6, and 7 who were all ERT-treated. 

Our results show that there was no clear pattern of cyclic biomarker variation observed in urine specimens of female participants during three cycles of ERT infusions. 

## 3. Discussion

The aim of the present study was to evaluate the variation of FD biomarkers, such as Gb_3_, lyso-Gb_3_, and related analogues, in urine specimens collected by seven participants diagnosed with FD (one untreated and six ERT-treated). Urine specimens were collected daily, at two different timepoints, for a duration of 42 days. The collection time (morning or evening urine specimens) and the administration of ERT infusions were evaluated as potential variability factors. A total of 84 urine specimens were collected at home by each study participant. Patient compliance was excellent as no urine specimen collection was missed. Two shipments with thawed urine specimens were received from participant no. 3 (day 26 to day 42). However, this was not an issue considering that Gb_3_ and lyso-Gb_3_ and related analogues are stable for at least 24 h at room temperature in urine specimens [7,9]. Urine specimens offer many advantages for biomarker analyses. Collection is safe and can be performed in a non-invasive way by patients, without the need for trained personnel such as phlebotomists. The analysis of spot urine specimens is more convenient compared to 24-h urine collection specimens, but results can vary according to the rate of urine production. Creatinine, a compound excreted at a constant rate in urine through glomerular filtration [19], is commonly used to normalize results, which are then compared with age- and gender-matched reference values. Gb_3_ normalization with creatinine might be arguable, considering that its presence in urine does not originate from glomerular filtration, but from the shedding of renal tubular cells and podocytes in urine [20]. Consequently, Gb_3_ normalization with membrane lipids such as sphingomyelin or phosphatidylcholine was previously investigated. Mills and colleagues [21] showed that the distribution of Gb_3_-to-sphingomyelin ratios was similar to the distribution of the Gb_3_-to-creatinine ratios. Whitfield and colleagues had the same conclusions regarding phosphatidylcholine [22]. Nevertheless, precautions should always be taken for the analysis of Gb_3_ in urine since urine specimens should be homogenized carefully, and filter papers impregnated with urine should be saturated and dried horizontally to avoid unequal distribution of Gb_3_ and creatinine molecules. 

Results obtained following the paired-sample *t*-tests show that the collection time might influence the biomarker levels in some cases. The measured values were generally more elevated in specimens collected in the evening, except for participant no. 3. Interestingly, there was no difference in lyso-Gb_3_ analogue (+14 Da) urinary levels depending on the collection time in all participants. This might indicate that this specific biomarker could be useful for long-term follow-up when it is not possible to collect urine specimens at a specific time. The results obtained following Levene’s test for equality of variances revealed that there was usually more biomarker level variability in the evening urine specimens. Specimens collected in the evening might be more prone to variations due to external factors such as physical activity, food intake, and water consumption. The results also showed that the concentrations of urinary lyso-Gb_3_ and Gb_3_ measured longitudinally were subject to more variability in the untreated patient than the measured concentrations of lyso-Gb_3_ analogues, according to RSDs (*n* = 42 days). A similar trend was observed in treated patients. This might indicate that the urinary levels of lyso-Gb_3_ analogues are more reliable and less variable for long-term follow-up and monitoring of FD patients than Gb_3_ and lyso-Gb_3_ itself. Regarding the variability of biomarker concentrations between ERT infusions, we initially hypothesized a cyclic biomarker variation with reduced biomarker levels immediately after infusion, followed by a slow increase over the rest of the 14-day cycle. A visual examination of Figure 3 and Figure 4 does not corroborate this initial hypothesis. Considering these results, urine specimens collected in the morning should be preferred over urine specimens collected in the evening for the quantification of FD biomarkers as part of a longitudinal evaluation. The results obtained do not suggest that urine specimens need to be collected at a specific time between two ERT infusions, since this was not a factor of variability in the present study. Urinary lyso-Gb_3_ analogues might be more reliable for longitudinal follow-up compared to lyso-Gb_3_ and Gb_3_, considering that some of these analogues showed less variability depending on the collection time than lyso-Gb_3_ or Gb_3_. It is noteworthy to mention that the analysis of lyso-Gb_3_ analogues is particularly relevant in urine, considering that lyso-Gb_3_ represents only approximately 2% of all lyso-Gb_3_-related species in this matrix. In comparison, lyso-Gb_3_ represents approximately 57% of all lyso-Gb_3_-related species in plasma [8,9].

## 4. Conclusions

According to the results obtained in this study, there is no cyclic variation of the urinary Fabry disease biomarkers (Lyso-Gb_3_ and analogues, and Gb_3_) normalized to creatinine between two ERT cycles. The diurnal variation observed for the urinary Fabry disease biomarkers normalized to creatinine might be attributed not only to the sphingolipid levels, but also to the variation in the creatinine levels. Nevertheless, the biomarker/creatinine ratios show less variation in urine specimens collected in the morning. Moreover, the lyso-Gb_3_ analogue/creatinine ratios show less variation than the lyso-Gb_3_/creatinine and Gb_3_/creatinine ratios. Finally, this study suggests that the urinary Fabry disease biomarker/creatinine ratios are more elevated in specimens collected in the evening except for the lyso-Gb_3_ analogue (+14 Da) which shows similar abundance at both collection times.

## 5. Materials and Methods

### 5.1. Ethics Approval

This multicenter research project was approved by the Research Ethics Board (REB) at the Faculty of Medicine and Health Sciences at the Centre Intégré Universitaire de Santé et de Services Sociaux de l’Estrie-Centre Hospitalier Universitaire de Sherbrooke (CIUSSSE-CHUS) (REB no 06-011, Approval date: 22 February 2006) and at the CIUSSS du Nord-de-l’Île-de-Montréal-Hôpital du Sacré-Coeur de Montréal (REB no 2014-1071, Approval date: 16 October 2014). All subjects provided informed consent for inclusion before their participation in the study. The study was conducted in accordance with the Declaration of Helsinki.

### 5.2. Recruitment of Participants

Seven FD patients who were confirmed either by demonstrating a marked enzyme deficiency of α-galactosidase A and/or via *GLA* gene mutation analysis. They were recruited from two centers in the province of Quebec: CIUSSSE-CHUS (Sherbrooke, QC, Canada) and CIUSSS du Nord-de-l’Île-de-Montréal-Hôpital du Sacré-Coeur de Montréal (Montreal, QC, Canada). Participant demographics are shown in Table 3. All participants, except one, were receiving ERT.

### 5.3. Urine Specimen Collection

Urine specimen collection was performed at home by participants. Each participant received the following urine collection kit: 86 conical centrifuge tubes in polypropylene (50 mL) divided into four Styrofoam racks, four pre-addressed shipping boxes, eight icepacks, parafilm, and four large plastic bags. Two urine specimens (identified as M and E) were collected each day for a duration of 42 days, representing three ERT cycles for treated patients. Specimen M was collected in the morning (before breakfast), and specimen E was collected in the evening, after dinner. The collection of the first urine specimen was performed when ERT-treated patients received their day-1 infusion. Parafilm was used to seal the cap of the tube after urine collection, and the tube was frozen immediately. Collected urine specimens (*n* = 25) were placed in a Styrofoam rack and inserted in a large plastic bag in a pre-addressed box containing two icepacks. Samples were picked up by a shipping courier and delivered to our laboratory in Sherbrooke, QC for analysis.

### 5.4. Analysis of Fabry Disease Biomarkers

FD biomarkers were analyzed as mentioned below using assays previously devised and validated in our laboratory. Intra-assay and inter-assay precisions were good with relative standard deviations (RSDs) ≤ 15%.

#### 5.4.1. Gb_3_

Total Gb_3_ was measured (total ion count (TIC)) for C16:0, C18:0, C20:0, C22:1, C22:0, C24:1, C24:0, and C24:OH isoforms simultaneously with creatinine in dried urine spots (DUS) as previously described [23,24]. Briefly, urine specimens were mixed carefully; then, 1 mL was deposited on a 5-cm-diameter disc of Whatman-GE 903 filter paper and dried completely at room temperature for at least 4 h. DUS extraction was performed by adding 4 mL of methanol to the filter paper disc and shaking for 1 h with an orbital shaker (300 rpm). Next, 10 µL of the extract was analyzed by high-performance liquid chromatography (HPLC) coupled to tandem mass spectrometry (MS/MS). The normal reference value was defined as ≤25 µg/mmol creatinine. Urine sample aliquots from an untreated Fabry male and an untreated Fabry female were used as high- and low-quality controls for this study. The intra- and interday precision values measured (RSD%) were respectively ≤12.5% and ≤11.3% for Gb_3_ and ≤10.1% and ≤7.0% for creatinine [24]. The intra- and interday accuracy values (Bias%) measured for Gb_3_ at a concentration of 3.75 µg/mL were respectively 4.3% and 7.9%.

#### 5.4.2. Lyso-Gb_3_ and Related Analogues

Lyso-Gb_3_ and seven related analogues were analyzed by ultra-performance liquid chromatography (UPLC) coupled to MS/MS, following solid-phase extraction with Oasis mixed-mode strong cation exchange (MCX) cartridges (Waters Corp., Milford, MA, USA), as previously described [9,25]. Normal reference values were 0 pmol/mmol creatinine (not detected) for lyso-Gb_3_ and the analogues (−28 Da), (−12 Da), (−2 Da), and (+14 Da). Normal reference values were 17, 14, and 55 pmol/mmol creatinine for the lyso-Gb_3_ analogues (+16 Da), (+34 Da), and (+50 Da), respectively. Urine sample aliquots from an untreated Fabry male and an untreated Fabry female were used as high- and low-quality controls for this study. For lyso-Gb_3_ and its seven analogues, the intra- and interday assay precision values (RSD%) were respectively ≤13.0% and ≤20.1%. The intraday and interday accuracy values (Bias %) for lyso-Gb_3_ at concentrations of 1500, 11,000, and 24,000 pM were <7.9% [9]. 

### 5.5. Statistical Analyses

Statistical analyses were performed using IBM SPSS Statistics version 24, while bar charts were done using GraphPad Prism version 8.2.1. Paired-sample *t*-tests were performed to evaluate if the mean difference of the measured biomarker concentration was significantly different from zero depending on the collection time. Shapiro–Wilk’s test and visual inspection of a normal quantile–quantile (Q–Q) plot were used to determine if the differences in biomarker levels between the two collection times were normally distributed. If not, a mathematical transformation was applied to obtain normally distributed data. Levene’s test for equality of variances was performed to evaluate if the variance of the measured biomarker levels was equal for both collection times over 42 days.

For all analyses, statistical significance was established at *p* ≤ 0.05, and the Holm–Šídák procedure was used for multiple comparisons [26]. Specimen E, day 15 from patient 1 was excluded from statistics for lyso-Gb_3_ + analogue results only, considering that the lyso-Gb_3_ internal standard (IS) was off (−48%) for this specimen, leading to falsely elevated results. Specimen E, day 34 from patient 7 was also excluded from statistics, considering that it was an extreme outlier (>3 interquartile ranges from the 75th percentile). This might be related to a low creatinine value in this sample (1.8 mmol/L), leading to unreliable results.

## Figures and Tables

**Table 1:** Paired-sample *t*-tests assessing mean differences in measured biomarker levels over 42 days in seven Fabry patients at two different collection time points (morning and evening). Means ± standard deviation are shown. For statistically significant results, *p*-values are highlighted in gray or in black if the highest mean concentration value was measured in the morning or the evening, respectively.

	Participant 1			Participant 2			Participant 3			Participant 4			Participant 5			Participant 6			Participant 7		
	M	E	*p*	M	E	*p*	M	E	*p*	M	E	*p*	M	E	p	M	E	p	M	E	p
**Gb_3_**	373 ± 128	468 ± 261	<0.050	28 ± 8	44 ± 23	**<0.001 ^†^**	12 ± 5	10 ± 3	<0.050	33 ± 9	38 ± 11	<0.050	20 ± 8	29 ± 12	**<0.001 ^†^**	49 ± 17	66 ± 52	0.059	4 ± 2	6 ± 4	0.060
**Lyso-Gb_3_**	188 ± 86	295 ± 94	**<0.001 ^†^**	59 ± 27	111 ± 61	**<0.001 ^†^**	22 ± 9	21 ± 7	0.525	62 ± 12	81 ± 27	<0.050	27 ± 11	34 ± 13	<0.050	82 ± 14	119 ± 36	**<0.001 ^†^**	12 ± 5	17 ± 6	**<0.001 ^†^**
**Lyso-Gb_3_ (−28)**	204 ± 46	228 ± 33	<0.050	23 ± 7	30 ± 7	**<0.001 ^†^**	14 ± 5	12 ± 4	<0.050	64 ± 8	55 ± 16	**0.001 ^†^**	13 ± 3	18 ± 6	**<0.001 ^†^**	42 ± 9	40 ± 12	0.224	5 ± 2	6 ± 3	0.208
**Lyso-Gb_3_ (−12)**	1620 ± 193	1576 ± 281	0.282	77 ± 17	77 ± 22	0.894	65 ± 15	53 ± 13	**<0.001 ^†^**	224 ± 33	226 ± 43	0.831	56 ± 14	50 ± 15	<0.050	99 ± 15	93 ± 30	0.224	50 ± 7	46 ± 9	<0.050
**Lyso-Gb_3_ (−2)**	585 ± 126	689 ± 95	**<0.001 ^†^**	35 ± 10	44 ± 11	**<0.001 ^†^**	32 ± 10	27 ± 9	<0.050	122 ± 15	118 ± 30	0.393	24 ± 5	33 ± 10	**<0.001 ^†^**	77 ± 10	92 ± 26	**0.001 ^†^**	9 ± 3	11 ± 5	0.135
**Lyso-Gb_3_ (+14)**	894 ± 138	855 ± 188	<0.050	24 ± 6	23 ± 6	0.105	26 ± 6	23 ± 6	<0.050	94 ± 14	93 ± 22	0.457	14 ± 3	14 ± 3	0.768	27 ± 5	24 ± 12	0.280	8 ± 2	9 ± 4	0.457
**Lyso-Gb_3_ (+16)**	5524 ± 1017	5246 ± 1351	<0.050	204 ± 43	206 ± 45	0.760	215 ± 39	182 ± 42	**<0.001 ^†^**	797 ± 109	800 ± 196	0.927	115 ± 27	110 ± 21	0.279	267 ± 40	283 ± 73	0.234	67 ± 11	72 ± 20	0.094
**Lyso-Gb_3_ (+34)**	2892 ± 440	2622 ± 532	0.001	121 ± 26	118 ± 28	0.527	125 ± 25	104 ± 26	**<0.001 ^†^**	315 ± 60	316 ± 98	0.797	80 ± 18	72 ± 13	<0.050	146 ± 25	142 ± 39	0.609	60 ± 11	65 ± 20	0.137
**Lyso-Gb_3_ (+50)**	4973 ± 711	4613 ± 913	<0.050	69 ± 15	68 ± 20	0.779	99 ± 20	77 ± 18	**<0.001 ^†^**	144 ± 27	144 ± 37	0.847	39 ± 9	34 ± 8	<0.050	70 ± 13	59 ± 20	<0.050	51 ± 7	50 ± 11	0.583

^†^ indicates that statistical significance was still valid after correction for multiple comparisons using the Holm–Šídák procedure (63 comparisons); M: morning collection; E: evening collection. All participants were under ERT except for participant no. 1 who was an ERT-naïve patient. Patients 1–4 were males, whereas patients 5–7 were females. For lyso-Gb_3_ analogues, the mass difference in Da compared to lyso-Gb_3_ is indicated in brackets.

**Table 2:** Levene’s test for equality of variances examining the influence of urine collection time on the variability of the measured biomarker levels over 42 days in seven Fabry patients. Relative standard deviations (RSDs) are shown (*n* = 42) for each collection time. For statistically significant results, *p*-values are highlighted in gray or in black if the highest variance was obtained in the morning or in the evening, respectively.

	Participant 1			Participant 2			Participant 3			Participant 4			Participant 5			Participant 6			Participant 7		
	%RSD			%RSD			%RSD			%RSD			%RSD			%RSD			%RSD		
	M	E	*p*	M	E	*p*	M	E	*p*	M	E	*p*	M	E	*p*	M	E	*p*	M	E	*p*
**Gb_3_**	34	56	0.190	30	51	**<0.001 ^†^**	44	31	0.101	27	28	0.089	38	40	0.495	35	79	<0.050	37	65	<0.050
**Lyso-Gb_3_**	46	32	0.813	46	55	<0.050	41	32	0.247	20	33	**<0.001 ^†^**	39	37	0.448	17	30	**<0.001 ^†^**	38	37	0.118
**Lyso-Gb_3_ (−28)**	23	15	0.126	30	25	0.614	33	32	0.170	13	29	<0.050	26	33	<0.050	21	31	0.137	33	53	<0.050
**Lyso-Gb_3_ (−12)**	12	18	0.156	22	29	0.227	23	24	0.562	15	19	0.538	26	29	0.889	15	32	**<0.001 ^†^**	14	19	0.738
**Lyso-Gb_3_ (−2)**	22	14	0.079	28	24	0.790	30	32	0.426	12	25	**<0.001 ^†^**	22	30	<0.050	14	28	**<0.001 ^†^**	32	48	<0.050
**Lyso-Gb_3_ (+14)**	15	22	0.277	23	26	0.696	22	25	0.708	14	24	0.189	20	24	0.903	18	49	**<0.001 ^†^**	22	42	**<0.001 ^†^**
**Lyso-Gb_3_ (+16)**	18	26	0.300	21	22	0.691	18	23	0.881	14	24	0.183	23	20	0.317	15	26	**<0.001 ^†^**	17	28	<0.050
**Lyso-Gb_3_ (+34)**	15	20	0.783	21	24	0.917	20	25	0.776	19	31	0.170	23	18	<0.050	17	27	<0.050	18	31	**<0.001 ^†^**
**Lyso-Gb_3_ (+50)**	14	20	0.285	21	30	0.150	20	24	0.308	18	26	0.536	23	22	0.056	18	34	0.063	14	22	<0.050

^†^ indicates that statistical significance was still valid after correction for multiple comparisons using the Holm–Šídák procedure (63 comparisons); M: morning collection; E: evening collection; RSD: relative standard deviation. For lyso-Gb_3_ analogues, the mass difference in Da compared to lyso-Gb_3_ is indicated in brackets.

**Table 3:** Participant demographics. n/a: not applicable; ERT: enzyme replacement therapy; eGFR: estimated glomerular filtration rate; CKD-EPI: Chronic Kidney Disease Epidemiology Collaboration (CKD-EPI) equation.

Patient No	1	2	3	4	5	6	7
Gender	M	M	M	M	F	F	F
Age (years)	30	28	40	36	49	57	67
Treatment status	No treatment	Replagal	Fabrazyme	Fabrazyme	Replagal	Replagal	Fabrazyme
Mutation	c.17_327del	c.612G > A	c.35_47del13	c.1241T > C	c.1042G > C	c.1241T > C	c.877C > A
ERT start date	n/a	2005-07-26	2008-09-30	2003-05-31	2009-01-14	2011-02-02	2003-04-15
Serum creatinine (µmol/L)	97	78	97	96	57	76	92
eGFR (CKD-EPI) (mL/min/1.73 m^2^)	91	116	83	87	105	76	56
Urinary proteins (g/24 h)	1.07	0.14	1.19	1.38	0.25	3.21	0.07

## Supplementary Materials

The following are available online at https://www.mdpi.com/1422-0067/21/17/6114/s1, Table S1. Raw study dataset; ERT: enzyme replacement therapy.

## Abbreviations

CIUSSSE-CHUS	Centre Intégré Universitaire de Santé et de Services Sociaux de l’Estrie-Centre Hospitalier Universitaire de Sherbrooke
DUS	Dried urine spots
ERT	Enzyme replacement therapy
FD	Fabry disease
Ga_2_	Galabiosylceramide
Gb_3_	Globotriaosylceramide
HPLC	High-performance liquid chromatography
LC-MS/MS	Liquid chromatography tandem mass spectrometry
Lyso-Gb_3_	Globotriaosylsphingosine
MCX	Mixed-mode strong cation exchange
MS/MS	Tandem mass spectrometry
REB	Research Ethics Board
RSD	Relative standard deviation
TIC	Total ion count
UPLC	Ultra-performance liquid chromatography
