# Supplementary figures and images for: Diurnal Variation of Urinary Fabry Disease Biomarkers during Enzyme Replacement Therapy Cycles
Source: Int J Mol Sci. 2020 Aug 25;21(17):6114. doi: 10.3390/ijms21176114 (PMC7503492; doi:10.3390/ijms21176114)

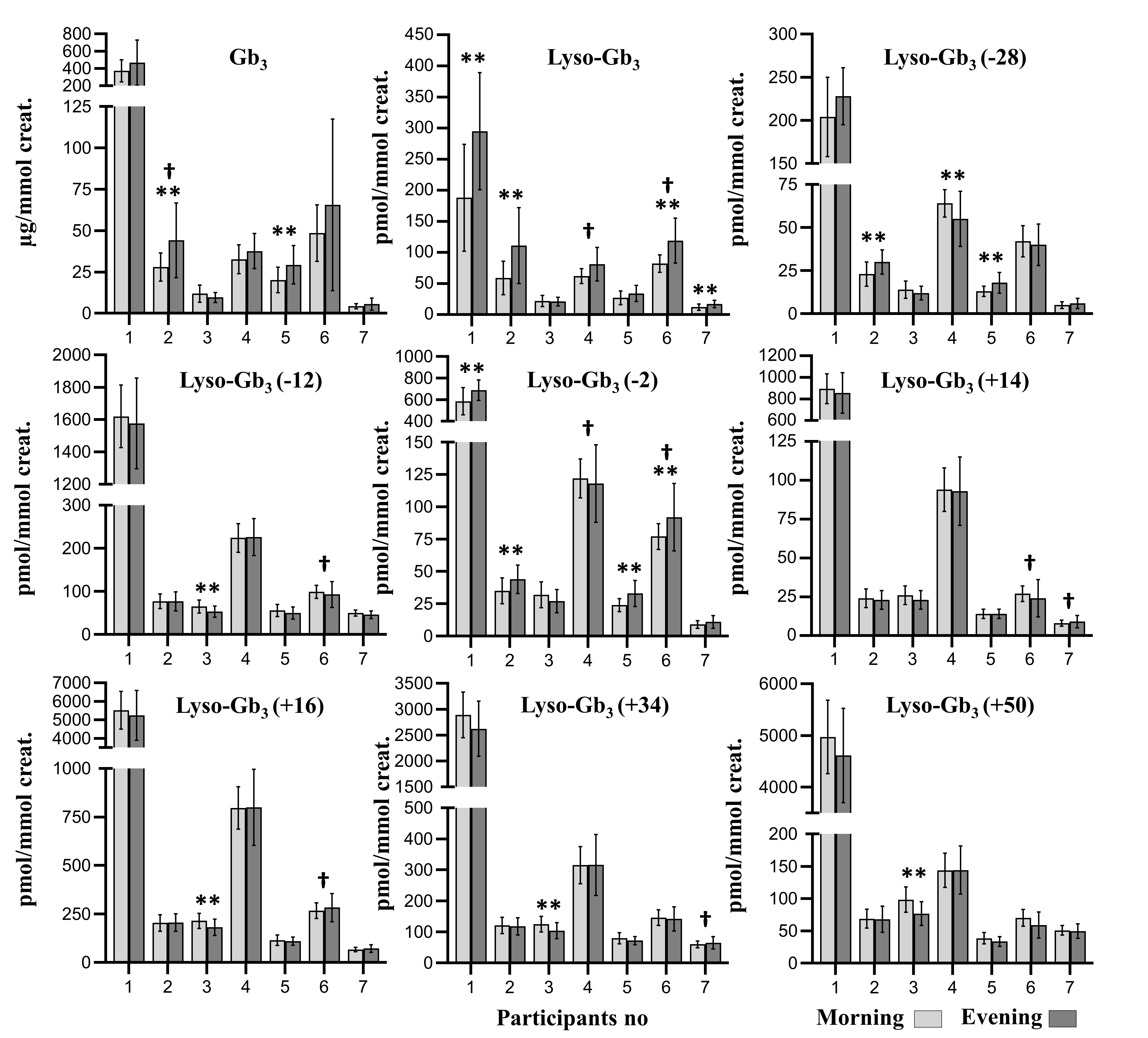

Supplement: Supplementary file 1 [file ijms-21-06114-s001.zip › Figure 1 - revision600.tif]

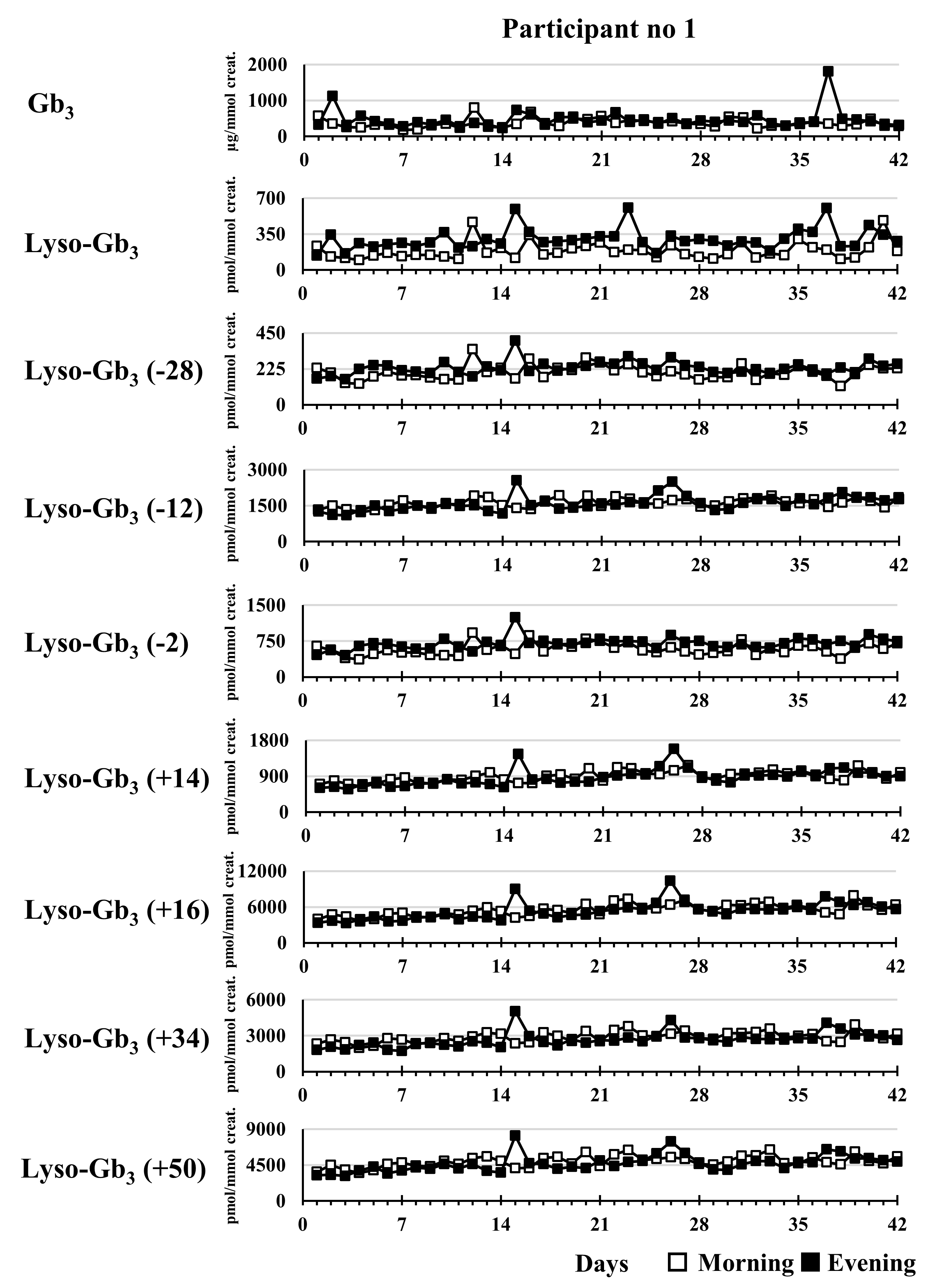

Supplement: Supplementary file 1 [file ijms-21-06114-s001.zip › Figure 2 - revision600.tif]

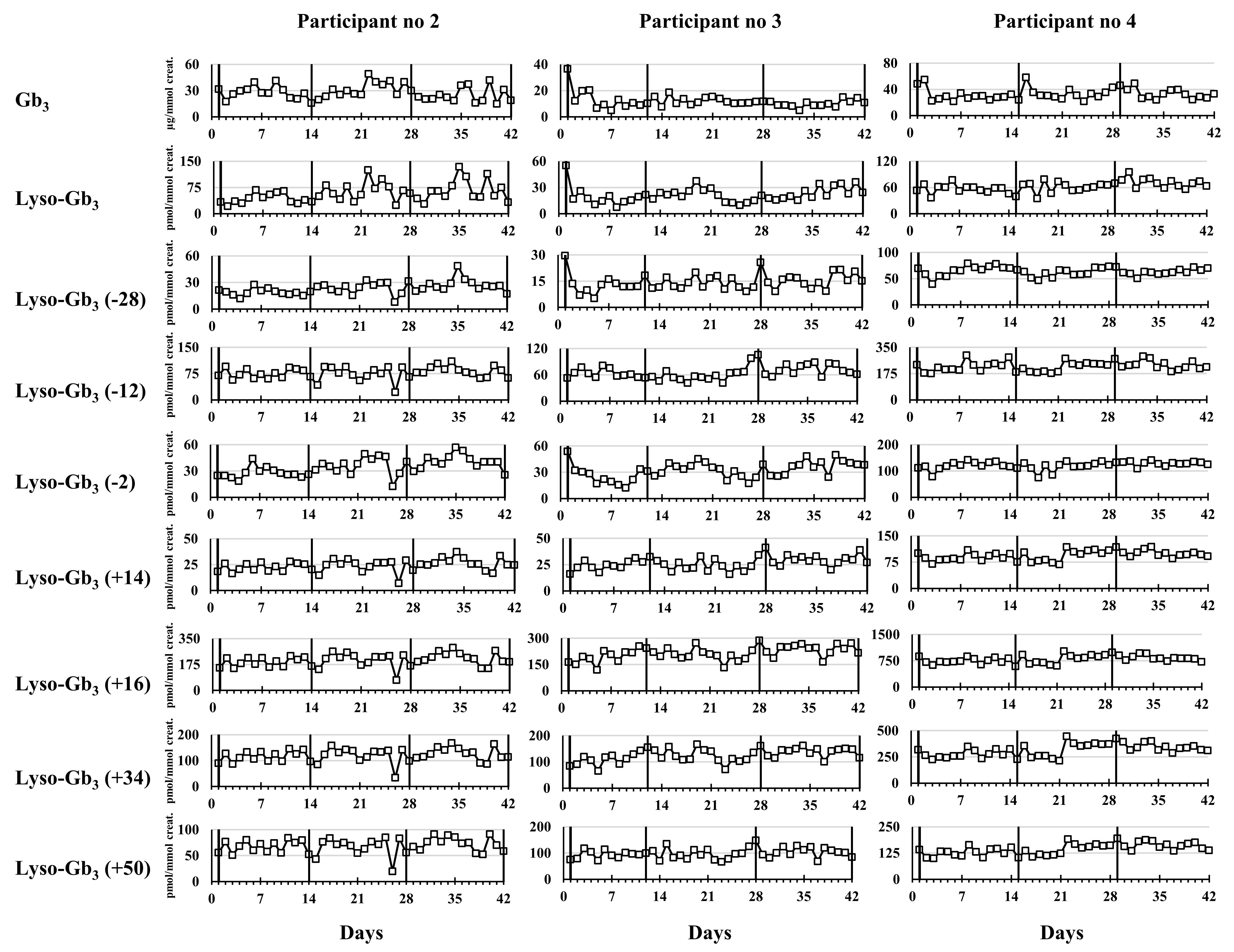

Supplement: Supplementary file 1 [file ijms-21-06114-s001.zip › Figure 3 - revision600.tif]

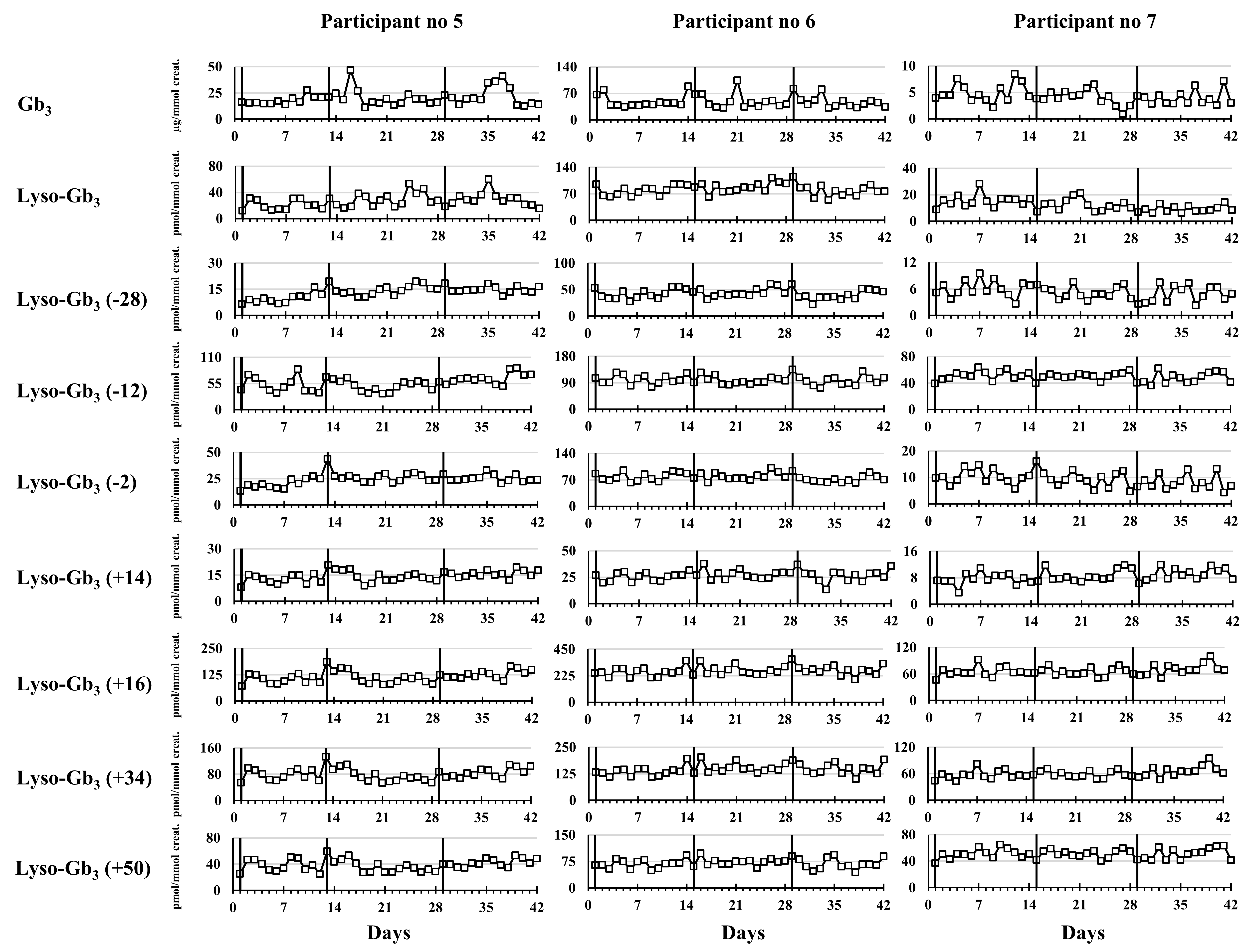

Supplement: Supplementary file 1 [file ijms-21-06114-s001.zip › Figure 4 - revision600.tif]
